# MRA-YOLOv8: A Network Enhancing Feature Extraction Ability for Photovoltaic Cell Defects

**DOI:** 10.3390/s25051542

**Published:** 2025-03-02

**Authors:** Nannan Wang, Siqi Huang, Xiangpeng Liu, Zhining Wang, Yi Liu, Zhe Gao

**Affiliations:** College of Information, Mechanical and Electrical Engineering, Shanghai Normal University, Shanghai 201418, China; 1000516012@smail.shnu.edu.cn (N.W.); 1000518969@smail.shnu.edu.cn (S.H.); 1000517848@smail.shnu.edu.cn (Z.W.); 1000552739@smail.shnu.edu.cn (Y.L.)

**Keywords:** photovoltaic cell defect detection, MBCANet, ResBlock, AMPDIoU, MRA-YOLOv8

## Abstract

To address the challenges posed by complex backgrounds and the low occurrence in photovoltaic cell images captured by industrial sensors, we propose a novel defect detection method: MRA-YOLOv8. First, a multi-branch coordinate attention network (MBCANet) is introduced into the backbone. The coordinate attention network (CANet) is incorporated to mitigate the noise impact of background information on the detection task, and multiple branches are employed to enhance the model’s feature extraction capability. Second, we integrate a multi-path feature extraction module, ResBlock, into the neck. This module provides finer-grained multi-scale features, improving feature extraction from complex backgrounds and enhancing the model’s robustness. Finally, we implement alpha-minimum point distance-based IoU (AMPDIoU) to the head. This loss function enhances the accuracy and robustness of small object detection by integrating minimum point distance-based IoU (MPDIoU) and Alpha-IoU methods. The results demonstrate that MRA-YOLOv8 outperforms other mainstream methods in detection performance. On the photovoltaic electroluminescence anomaly detection (PVEL-AD) dataset, the proposed method achieves a *mAP*_50_ of 91.7%, representing an improvement of 3.1% over YOLOv8 and 16.1% over detection transformer (DETR). On the SPDI dataset, our method achieves a *mAP*_50_ of 69.3%, showing a 2.1% improvement over YOLOv8 and a 6.6% improvement over DETR. The proposed MRA-YOLOv8 also exhibits great deployment potential. It can be effectively integrated with drone-based inspection systems, allowing for efficient and accurate PV plant inspections. Moreover, to tackle the issue of data imbalance, we propose generating synthetic defect data via generative adversarial networks (GANs), which can supplement the limited defect samples and improve the model’s generalization ability.

## 1. Introduction

The 21st century can be regarded as the ‘era of global energy’, with society exhibiting an unprecedented focus on energy. Driven by factors such as the rapid post-pandemic economic rebound, the energy market began to tighten after 2021. Consequently, many households faced economic hardship, some factories had to reduce or stop production, and economic growth stagnated, leading some countries into severe recession. The International Energy Agency described this as ‘the first truly global energy crisis’. The global push toward clean energy has reached a critical juncture. In China, solar energy, a clean and sustainable resource, is emerging as a key focus in energy development. China has very rich solar energy resources, with annual radiation exceeding 5000 $MJ/m^{2}$. Therefore, abundant solar energy resources provide feasibility for China’s transition toward clean energy [1,2]. The most common solar power generation technologies currently utilize photovoltaic (PV) panels, which consist of solid-state PV cells that convert light energy into electricity when exposed to sunlight.

However, during production and usage, the energy efficiency of PV cells is often limited by defects such as cracks, black core, and star cracks [3,4,5] caused by mechanical stress, thermal stress, and human factors. These defects reduce the performance and lifespan of PV cells, thereby affecting their power generation efficiency [6]. Defect detection methods are used for the condition monitoring of PV systems and thus assist in predictive maintenance. In recent years, researchers have designed various methods for defect detection in PV cells. Traditional fault detection and diagnosis (FDD) methods [7] typically detect anomalies based on predefined rules or thresholds, relying on physical models and the knowledge of domain experts. These methods require manual feature engineering design and offer strong interpretability and adaptability. However, these methods also have certain drawbacks, including a high dependency on expert knowledge, complex feature extraction, limited generalization capability, insufficient real-time performance, and sensitivity to data imbalance. Along with the rise of deep learning and data-driven methods, many limitations of traditional FDD approaches are being overcome, providing more automated, accurate, and efficient solutions for FDD [8]. Meanwhile, the development of deep learning technology has brought new opportunities for fault diagnosis in PV systems. By automatically learning features and recognizing patterns from complex data, deep learning has improved the accuracy and robustness of fault diagnosis. Firstly, deep learning algorithms possess powerful feature learning capabilities, enabling them to automatically extract complex feature representations from raw data without the need for manually designed feature extractors. Secondly, deep learning models such as deep neural networks can effectively handle the nonlinear and high-dimensional data in PV systems, including complex patterns and interactions, thereby improving the accuracy of fault diagnosis. Additionally, deep learning technology is able to integrate multi-source information and has good generalization capabilities, allowing it to adapt to system changes under different environmental conditions. It is worth noting that deep learning can also automate the fault diagnosis process, reducing the reliance on expert knowledge. Additionally, it can handle imbalanced datasets and employ strategies such as incremental learning and transfer learning to identify complex fault patterns and enhance accuracy. These advantages make deep learning a promising and powerful tool in the field of fault diagnosis for PV systems [9].

Deep learning-based FDD algorithms possess a distinct advantage over traditional methods [10]. This renders intelligent FDD techniques significantly more effective. While the advent of deep learning has propelled diagnostic research forward, it remains a nascent field necessitating further extensive investigation. Numerous challenges persist in the application of deep learning to defect detection [11].

In PV systems, the distribution of different categories of data is not balanced. To address FDD problems under these conditions, a wide range of solutions can be created, including focal loss functions, data-level undersampling, and oversampling methods. These can then be applied as inputs to existing DL-based methods. Additionally, in practical applications, the collected data may contain noise, missing values, or imbalanced data types, all of which can affect the performance of deep learning models. Accordingly, it is vital to filter measurement noise.

Due to the smaller area covered by small-scale targets, their positions lack diversity, making the generalization of small target detection during validation more challenging. Additionally, the complexity of the background, including varying degrees of occlusion, blurriness, and incompleteness, further increases the difficulty of detecting small-scale targets. Therefore, the main challenge lies in enhancing the feature extraction capabilities of deep learning networks to ensure the accurate identification of defects in PV cells. To address these challenges, we propose MRA-YOLOv8, an innovative defect detection method for PV cells. We introduce an MBCANet to the backbone to enhance feature extraction capabilities and reduce the noise impact of background information on the detection task. In the neck, ResBlock is incorporated to utilize its finer-grained multi-scale features for better feature extraction from complex backgrounds, thereby improving model robustness. In the head, we integrate alpha-minimum point distance-based IoU (AMPDIoU) to enhance the accuracy and robustness of small target detection. The results indicate that MRA-YOLOv8 outperforms existing detection methods. This superiority not only demonstrates the effectiveness of MRA-YOLOv8 but also indicates that with higher accuracy, it can detect defects more precisely. Fewer defective modules are overlooked, reducing maintenance and replacement costs. It also improves the stability and reliability of the PV system, lowering the risk of energy production interruptions and indirectly reducing energy waste. Additionally, more accurate detection means fewer normal modules are scrapped, saving raw materials and production costs. This also avoids the energy used to re-manufacture these modules. On the production line, rapid and accurate detection is key. MRA-YOLOv8 can quickly inspect large numbers of PV modules, improving efficiency and smoothing the production process. This reduces time costs and potential energy waste caused by delays. In real-world applications, our MRA-YOLOv8 model shows great potential for deployment, particularly when combined with drone-based inspection systems. The model is lightweight, allowing it to run efficiently on drones with limited computing power. This enables real-time processing of high-resolution images captured by the drones’ cameras during flight. Even with the image jitter and rapid changes in lighting that are common in drone imaging, the robust design of the model ensures accurate and stable detection of defects. These advantages are highly compatible with the requirements of PV power plant inspections. Drones equipped with high-definition cameras fly along preset routes over the power plants. The MRA-YOLOv8 model analyzes the captured images, either directly on the drone or in a ground control center via real-time data transmission. It can quickly identify various defects in PV modules, such as hot spots, hidden cracks, and breakages, to improve the reliability and efficiency of distributed PV power systems.

The remainder of this paper is structured as follows. Section 2 reviews defect detection methods, spanning traditional techniques to deep learning advancements, particularly YOLO-based solutions. Section 3 details the proposed MRA-YOLOv8 framework, systematically elaborating the design of an MBCANet in the backbone, ResBlock in the neck, and AMPDIoU in the detection head. Section 4 introduces the experimental configurations, covering dataset characteristics, implementation protocols, and evaluation metrics used in this study. Section 5 presents extensive experimental validations through ablation studies, comparative analyses with state-of-the-art methods, and visualizations of detection results. Finally, Section 6 concludes this study by summarizing the key contributions and proposing future research directions to address the current limitations.

## 2. Related Work

In recent years, significant efforts have been devoted to detecting defects in PV cells. However, conventional visual inspections and camera-based imaging techniques often fail to accurately identify latent defects such as cracks, scratches, broken corners, and black cores. To address this issue, researchers have proposed solutions primarily involving imaging-based detection and deep learning-based detection methods [12].

### 2.1. Traditional Photovoltaic Cell Detection Techniques

Currently, the mainstream defect detection method used in China is electroluminescence (EL) detection [13]. When a solar cell module is energized, it emits infrared light. The weaker the intensity of the infrared light, the more pronounced the internal crystal grain boundaries and external defects become. EL images can detect internal microcracks and various other defects. However, challenges such as data imbalance, varying defect scales, and significant detection lag pose great difficulties for the detection task, leading to material and labor waste. Photoluminescence (PL) defect detection technology uses light with a wavelength greater than the bandgap of the silicon wafer to excite carriers in the silicon. When the light source is removed, the excited electrons, which are in a metastable state, return to the ground state within a short period. The photons released during this process are captured by a charge-coupled device (CCD) camera, resulting in a radiative recombination image of the silicon wafer. PL detection technology can be used to analyze various process stages, including diffusion, etching, and cell manufacturing. Compared to EL, it can identify issues in the production process more quickly and effectively, providing a reliable guarantee of product quality. The difference between EL and PL detection technologies lies in their carrier injection methods: EL injects carriers through the cell’s electrodes, whereas PL uses a light injection method to inject carriers. By combining both techniques during the detection process, a more comprehensive identification of cell defects can be achieved.

### 2.2. Thermography-Based Photovoltaic Cell Detection Technology

Another commonly used technology for detecting PV resistance defects is infrared thermography [14]. Infrared thermography uses photoelectric technology to detect specific wavelength signals of an object’s thermal radiation. These signals are then converted into images and graphics discernible by human vision, and further temperature values can be calculated. For data acquisition, only an infrared camera is needed, without the need for additional equipment or interruption of the PV system’s operation [15]. He et al. [16] proposed eddy current thermography, which utilizes Faraday’s law of electromagnetic induction. If a defect is present in the test workpiece, the intensity and distribution of the eddy current field are altered, causing a change in the coil impedance. By detecting this change, the presence of defects can be determined, thereby constructing the eddy current distribution for eddy current pulse thermography. Eddy current testing is commonly used for the high-speed inspection of conductive materials. It does not require any contact between the test piece and the sensor, making it suitable for defect detection in PV cells. Similar to eddy current thermography, lock-in thermography (LIT) [17] can also achieve non-contact surface temperature measurement. This technique offers the benefits of both infrared heat wave technology and digital blocking technology, lowering the requirements for heat uniformity and surface irradiance. Consequently, the signal-to-noise ratio, detection sensitivity, and detection capability are significantly improved. This technology is often used to detect parallelism defects in modules caused by cutting or laser scratching during the PV cell manufacturing process. Additionally, Umar et al. [18] proposed ultrasonic infrared thermography. In this technique, an ultrasonic generator generates electrical signals and transmits them to an ultrasonic gun. When these ultrasonic waves encounter defects such as cracks or exfoliations, the particles in the medium are subjected to vibratory forces under the excitation of the ultrasonic waves. When the interfaces of the two media collide, the resulting friction leads to an energy conversion that results in a significant temperature increase at the defect site and its neighborhood. This method has the advantage of selective heating and the ability to detect cracks in complex workpieces. However, although the aforementioned imaging-based defect detection techniques are relatively easy to implement, their detection accuracy is often unsatisfactory. For example, in terms of recall, infrared thermography may miss some small-scale defects due to its limited resolution, resulting in a relatively low recall value. In contrast, deep learning-based methods can learn complex features from a large amount of data, potentially achieving higher recall. In terms of mAP, the imaging-based techniques usually have a lower mAP value because they may not be able to accurately detect defects in various scenarios. Deep learning-based methods, with their powerful feature extraction and classification capabilities, are more likely to achieve the desired level of accuracy. Therefore, deep learning methods are needed to achieve the desired level of accuracy.

### 2.3. Deep Learning-Based Photovoltaic Cell Detection Techniques

Over the last few years, with the advancement of artificial intelligence technology, the defect detection of PV panels has gradually begun to be applied in combination with deep learning [19]. Deep learning image detection algorithms achieve autonomous extraction and recognition of target feature parameters by learning from large amounts of labeled image data. Through multiple iterations of training and optimization of learning weights, these algorithms significantly enhance detection performance. The key to applying deep learning in defect detection lies in considering FDD. The effectiveness of the detection method depends heavily on the quality of the extracted features. Therefore, the choice of models and tools is crucial. Defect detection based on convolutional neural networks (CNNs) is rapidly advancing. CNNs improve upon traditional neural networks by extracting features through multiple layers, enabling precise target detection [20,21]. Detection based on CNNs can maintain the translational invariance of extracted features under the conditions of random strong magnetic interference and high temperatures [22], thereby enhancing the robustness of diagnostic algorithms. Additionally, it can learn and detect various patterns present in EL images, enabling the detection of different kinds of PV defects, thereby preventing potential hazards. The cumulative effect of CNNs has improved the defect detection accuracy, thereby enhancing the durability and performance of PV modules [23]. Manohar et al. [24] proposed a protection scheme for fault detection/classification based on a deep learning approach using CNNs. When handling complex datasets, this method extracts features from images using convolutional and pooling layers and then classifies or recognizes these features through fully connected layers. This approach reduces computational complexity and costs. By using the probability distribution function to model irradiance levels over a period of time, the uncertainty of irradiance is accounted for. The inclusion of spatio-temporal distribution functions in the spatial data-based CNNs framework can improve the mapping between multidomain data and microgrid states. However, achieving generalization with CNNs in PV cell detection is challenging. Differences in installation locations result in varying levels of sunlight intensity and pollution. The training models for CNNs are often too singular, lacking the adaptability to recognize defects in different types of solar panels. Chen et al. [25] proposed an improved anomaly detection method based on Faster R-CNN for electroluminescence imaging of surface defects in PV cells. This approach enables more efficient analysis of crack defects in complex scenarios. Su et al. [26] proposed BAF-Detector. This approach enhances the network’s robustness to scale variations, resulting in superior performance in multi-scale defect detection tasks. Some scholars have applied the classical CNN (LeNet-5) to defect detection in solar panels. After discovering that the classification performance was suboptimal, TensorBoard was used for visualization to improve the network structure and hyperparameters.

### 2.4. Application of YOLO in Photovoltaic Cell Detection

YOLO algorithms, known for their comprehensive performance, have become widely used frameworks in object detection [27] and are gradually being applied to PV cell defect detection. Improvements in the detection accuracy of the YOLO series include the addition of multiple attention mechanisms and supplementary loss functions. Li et al. [28] achieved good accuracy in multi-scale target detection for PVpanel defects by using cross-stage partial (CSP) Bottleneck modules and incorporating a small object prediction head in YOLOv5. Zhang and Yin [29] developed a system using an improved YOLOv5 algorithm, integrating deformable convolutions to the CSP module and incorporating the ECA-Net attention mechanism. This enhancement significantly improved the model’s feature extraction capability. Additionally, researchers enhanced the model’s ability to detect minor defects, thereby improving target detection accuracy across different scales. MixUp and Mosaic data augmentation techniques helped their model achieve higher accuracy. Lu et al. [30] proposed the addition of adaptive spatial feature fusion to the original feature fusion structure of YOLOv5. This improved model enhances accuracy in detecting PV cell defects in EL images, and test time augmentation also significantly improves performance. Phan et al. [31] designed a new algorithm for defect detection in PV cell EL images, aiming to enhance YOLO’s performance by optimizing the learning rate and model parameters. However, the model’s accuracy in detecting defects across various categories and scales still requires improvement. To achieve more precise defect detection in PV cells, the MRA-YOLOv8 network is introduced in this study. We incorporate an MBCANet into the backbone, integrate ResBlock into the neck, and apply AMPDIoU to the head.

## 3. Methods

In recent years, the YOLO series has seen rapid development in the field of object detection. The YOLOv8 model [32], one of the latest iterations in this series, not only retains the computational efficiency of its predecessors but also deeply optimizes the network backbone architecture and feature extraction methods. As a result, this model is well suited for the task of detecting defects in PV cells, reducing the waste of human and material resources, and minimizing the time costs associated with manual identification in actual production processes.

**Figure 1:**
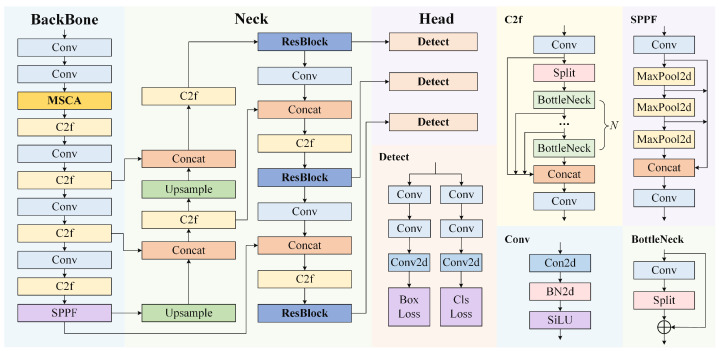
Architecture of the MRA-YOLOv8 network. Compared to YOLOv8, the proposed network integrates an MBCANet into the backbone, incorporates ResBlock into the neck, and improves the loss function in the detection component.

However, due to the complex backgrounds and the low proportion of defect feature information in PV cell images, using the YOLOv8 model alone is insufficient to achieve a high level of accuracy. To address this issue, we propose the MRA-YOLOv8 network, with its architecture illustrated in Figure 1.

First, we introduce a multi-branch coordinate attention network (MBCANet) into the backbone. The inclusion of a CANet [33] helps to reduce the noise impact of background information on the detection task, while the use of multiple branches enhances the model’s feature extraction capability. Second, we integrate an effective residual [34] module (ResBlock) into the neck. This module offers finer-grained multi-scale features, which can better extract features from complex backgrounds, thus improving the model’s robustness. Finally, AMPDIoU is incorporated into the detect block of the head. This loss function enhances the accuracy and robustness of small object detection by integrating the methods of minimum point distance-based IoU (MPDIoU) and Alpha-IoU.

### 3.1. MBCANet

PV cell defects are small and occupy a minimal amount of information in the entire image, with complex backgrounds and excessive interference information. To improve the accuracy of small object detection and reduce the interference of irrelevant information, we introduce the MBCANet into the backbone to adaptively focus on detailed information related to small objects, decreasing the attention to other information. The MBCANet contains multiple branches, each focusing on information of different sizes. We insert coordinate information into the channel attention of each branch. While conventional channel attention mechanisms use pooling operations to transform tensor-form features into vector-form features, we split the channel attention into two parts, encode features in each part along different directions, and then merge the two parts. This method allows us to obtain long-distance dependencies in one direction and retain coordinate information in the other. Additionally, a residual module is introduced to address the vanishing gradient problem [35] during gradient descent.

#### 3.1.1. Multi-Branch Design

**Figure 2:**
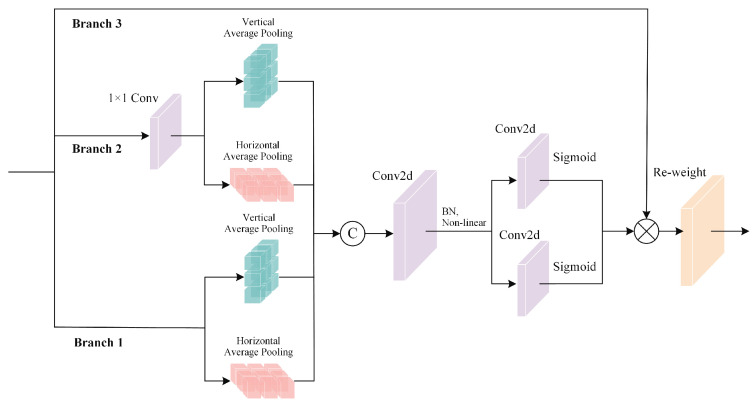
Structure of the multi-branch coordinate attention network (MBCANet). This network integrates both multi-branch architecture and a CANet.

As shown in Figure 2, the network structure of the MBCANet incorporates a residual block [36] with three branches. The first branch directly connects to the CANet, the second branch, after passing through a convolutional layer, connects to the CANet, and the third branch performs element-wise multiplication with the outputs of the first two branches. This design aims to address the vanishing gradient problem during gradient descent, ensuring a more stable and efficient gradient flow. Furthermore, this multi-branch structure aids in capturing features at different scales, thereby enhancing the network’s representational capacity and robustness.

#### 3.1.2. CANet Design

A CANet is a lightweight attention mechanism that leverages the coordinate information of bounding boxes and applies weights to enhance the model’s focus on critical feature points.

First, due to the difficulty of preserving coordinate information during the embedding stage, the CANet employs a global pooling method. Performing pooling operations separately in the horizontal and vertical directions ensures that channel attention retains long-range dependencies in both dimensions. Therefore, the output can be expressed as

(1) $$Z_{c}^{h}\left(h\right)=\frac{1}{W}\sum_{i=0}^{W}x_{c}\left(h,i\right),$$

(2) $$Z_{c}^{w}\left(w\right)=\frac{1}{H}\sum_{j=0}^{H}x_{c}\left(j,w\right),$$

where *C* represents the number of channels in the input image, while *H* and *W* denote its height and width, respectively. $Z_{c}^{h}$ and $Z_{c}^{w}$ represent the results of global pooling in the height and width directions, respectively.

After performing global pooling, we concatenate the feature vectors from these two directions and then apply convolution, batch normalization, and a nonlinear activation function:

(3) $$f=\sigma (F_{1}(Z^{h},Z^{w})),$$

where *F*_1_ denotes the convolution operation, *σ* represents the nonlinear activation function, and *f* is the feature map processed by convolution and the activation function.

After batch normalization and the nonlinear activation function, the feature map *f* is split into two parts, which are then convolved in the horizontal and vertical directions to induce attention. Subsequently, these feature maps *f* are combined with the Sigmoid function to form the attention vector, which can be expressed as follows:

(4) $$g^{h}=\sigma (F_{h}(f^{h})),$$

(5) $$g^{w}=\sigma \left(F_{w}\left(f^{w}\right)\right),$$

where $F_{h}$ and $F_{w}$ represent the convolution operations in the horizontal and vertical directions, respectively, while $g^{h}$ and $g^{w}$ denote the attention weights in the horizontal and vertical directions, respectively.

Finally, the output of the CANet is given by

(6) $$y_{c}\left(i,j\right)=x_{c}\left(i,j\right)\times g_{c}^{h}\left(i\right)\times g_{c}^{w}\left(j\right),$$

where $y_{c}$ is the output feature map, and $x_{c}$ is the input feature map.

The CANet focuses not only on the interactions between channels but also on the positional interactions within each channel. Compared to other attention mechanisms, it more effectively captures spatial relationships, thereby enhancing the model’s understanding of images and improving accuracy.

### 3.2. ResBlock

In the neck layer, we introduce the ResBlock module for multi-scale feature extraction. This is a compound structure that includes multiple Conv, Bottleneck, and Squeeze components. Conv combines convolution operations [37], batch normalization [38], and the Sigmoid activation function [39]. Batch normalization helps speed up training, improve generalization, and reduce internal covariate shift. The Sigmoid activation function introduces nonlinearity, which helps the network capture complex features. The Bottleneck uses an expansion factor to reduce the number of channels in the intermediate layers, significantly reducing the number of parameters and the computational burden while extracting deeper features through the deep fusion of two convolutional layers. Additionally, residual connections allow the network to maintain training stability while increasing depth by passing more gradients through skip connections, helping to address the vanishing gradient problem in deep networks. Squeeze [40] uses a 1 × 1 convolution kernel to reduce the number of channels, aiming to decrease the number of parameters and computational load while preserving important features.

The ResBlock module has specific data flow splitting and merging strategies that demonstrate significant advantages over traditional neck designs like PANet. Unlike PANet’s fixed-scale feature pyramid structure, ResBlock employs dynamic channel splitting, where input data are adaptively divided into multiple paths based on feature characteristics, enabling more flexible multi-scale processing. Each path handles different feature subsets, allowing the extraction of information at different levels and enhancing the network’s comprehensive understanding of features. This parallel processing approach contrasts with PANet’s sequential top-down and bottom-up propagation, enabling simultaneous extraction of both local details and global context. Ultimately, the outputs of these paths are merged through concatenation, which fuses features from different perspectives, improving feature representation. This hierarchical fusion mechanism outperforms PANet’s simple feature addition by preserving richer multi-scale interactions. Thus, through convolution kernels of different scales and multi-level feature processing, the network can adapt to feature extraction at various scales, which is particularly important for handling input images of different sizes.

**Figure 3:**
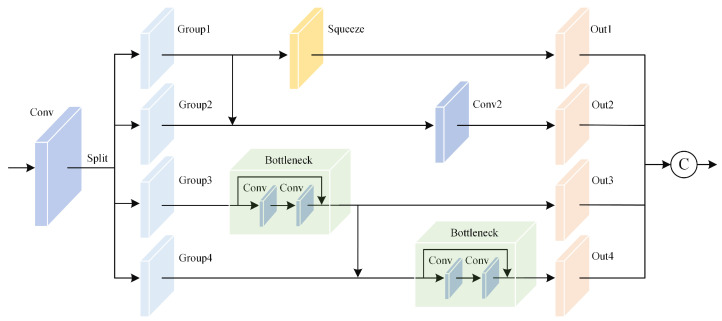
Structure of the residual module ResBlock. This architecture incorporates the concepts of both multi-branch design and residual connections.

The specific network structure of the ResBlock module is shown in Figure 3. The input features first pass through a 1 × 1 convolution kernel and are then divided into four groups according to the number of channels, with each feature subset having 1/4 of the channels. Specifically, the features that pass through the 1 × 1 convolution kernel enter Out1 after passing through a Squeeze operation. Group1 and Group2 are merged and then pass through another 1 × 1 convolution kernel to enter Out2. Group3 passes through a Bottleneck and then splits into two branches: one branch enters Out3 directly, while the other merges with Group4 and then passes through another Bottleneck to enter Out4. When each feature split group passes through the Bottleneck, the output will have a larger receptive field than the original group. The ResBlock module typically uses a multi-scale approach for splitting and processing, which helps in extracting both local and global information simultaneously. Finally, all of the feature splits are concatenated and passed through a 1 × 1 convolution to achieve better fusion of information from different scales. Additionally, using the Squeeze operation reduces the dimensions of some feature maps, which not only lowers the computational burden but also helps mitigate the overfitting problem.

### 3.3. AMPDIoU

We introduce AMPDIoU into the detection block of the head to enhance the accuracy and robustness of small object detection. In object detection, the predicted bounding box’s width and height values often differ significantly from those of the ground truth box. Previous loss functions could not effectively optimize this situation. The MPDIoU loss function [41] effectively addresses this issue. We set the coordinates of the ground truth and predicted bounding boxes as ${(x}_{1}^{gt},x_{2}^{gt},y_{1}^{gt},y_{2}^{gt})$, $\left(x_{1}^{prd},x_{2}^{prd},y_{1}^{prd},y_{2}^{prd}\right)$, respectively.

(7) $$x_{0}=\left(max\left(x_{2}^{gt},x_{2}^{prd}\right)-min\left(x_{1}^{gt},x_{1}^{prd}\right)\right),$$

(8) $$y_{0}=\left(max\left(y_{2}^{gt},y_{2}^{prd}\right)-min\left(y_{1}^{gt},y_{1}^{prd}\right)\right),$$

(9) $$\left|C\right|=x_{0}\times y_{0},$$

where $x_{0}$ and $y_{0}$ represent the width and height of the smallest enclosing rectangle that covers both the predicted and ground truth boxes, respectively. $\left|C\right|$ represents the area of the smallest enclosing rectangle that covers both the predicted and ground truth boxes.

(10) $$x_{c}^{gt}=\frac{x_{1}^{gt}+x_{2}^{gt}}{2},\;y_{c}^{gt}=\frac{y_{1}^{gt}+y_{2}^{gt}}{2},$$

(11) $$x_{c}^{prd}=\frac{x_{1}^{prd}+x_{2}^{prd}}{2},\;y_{c}^{prd}=\frac{y_{1}^{prd}+y_{2}^{prd}}{2},$$

where $\left(x_{c}^{gt},y_{c}^{gt}\right)$ and $\left(x_{c}^{prd},y_{c}^{prd}\right)$ represent the center coordinates of the ground truth and predicted bounding boxes, respectively.

(12) $$A^{prd}=\left(x_{2}^{prd}-x_{1}^{prd}\right)\times \left(y_{2}^{prd}-y_{1}^{prd}\right),$$

(13) $$A^{gt}=\left(x_{2}^{gt}-x_{1}^{gt}\right)\times \left(y_{2}^{gt}-y_{1}^{gt}\right),$$

where $A^{prd}$ and $A^{gt}$ represent the areas of the ground truth and predicted bounding boxes, respectively.

(14) $$d_{1}^{2}={\left(x_{1}^{prd}-x_{1}^{gt}\right)}^{2}+{\left(y_{1}^{prd}-y_{1}^{gt}\right)}^{2},$$

(15) $$d_{2}^{2}={\left(x_{2}^{prd}-x_{2}^{gt}\right)}^{2}+{\left(y_{2}^{prd}-y_{2}^{gt}\right)}^{2},$$

where $d_{1}^{2}$ and $d_{2}^{2}$ denote the Euclidean distance between the centers of the ground truth and predicted bounding boxes.

(16) $$x_{1}^{I}=max\left(x_{1}^{prd},x_{1}^{gt}\right),\;x_{2}^{I}=min\left(x_{2}^{prd},x_{2}^{gt}\right),$$

(17) $$y_{1}^{I}=max\left(y_{1}^{prd},y_{1}^{gt}\right),\;y_{2}^{I}=max\left(y_{2}^{prd},y_{2}^{gt}\right),$$

(18) $$I=\left\{\begin{array}{c} (x_{2}^{I}-x_{1}^{I})\times (y_{2}^{I}-y_{1}^{I}),\;\;\;\;\;\;if\;x_{2}^{I}>x_{1}^{I},\;y_{2}^{I}>y_{1}^{I}\\ 0,\;\;\;\;\;\;\;\;\;\;\;\;\;\;\;\;\;\;\;\;\;\;\;\;\;\;\;\;\;\;\;\;\;\;\;\;\;\;\;\;\;\;\;\;\;\;\;\;\;\;\;\;\;\;\;\;\;\;\;otherwise \end{array}\right.,$$

where ($x_{1}^{I}$, $x_{2}^{I}$, $y_{1}^{I}$, $y_{2}^{I}$) represent the coordinates of the intersection rectangle between the predicted and ground truth bounding boxes. *I* represents the intersection area between the ground truth and predicted bounding boxes.

(19) $$IoU=\frac{1}{U},$$

(20) $$U=A^{gt}+A^{prd}-I,$$

(21) $$MPDIoU=IoU-\frac{d_{1}^{2}}{h^{2}+w^{2}}-\frac{d_{2}^{2}}{h^{2}+w^{2}},$$

(22) $$L_{MPDIoU}=1-MPDIoU,$$

where *IoU* represents the intersection over union, and *U* represents the union area of the ground truth and predicted bounding boxes. *MPDIoU* is the modified *IoU* metric, and $L_{MPDIoU}$ is the corresponding loss function.

Additionally, we incorporate the Alpha-IoU method [42] into the MPDIoU loss function. The Alpha-IoU function provides more precise boundary regression, where α is a tunable hyperparameter that allows for the flexible realization of different levels of bounding box regression [43]. In our implementation, we set α = 1.2, as this value increases the loss and gradient for high *IoU* targets, thereby enhancing the precision of bounding box regression and improving the robustness of small object detection.

(23) $$L_{IoU}=1-IoU\Rightarrow L_{\alpha -IoU}=1-{IoU}^{\alpha },$$

where $L_{IoU}$ is the Alpha-IoU loss function, and α is a hyperparameter that controls the sensitivity of the loss function to different IoU values.

Thus, our bounding box regression loss function is defined as follows:

(24) $$L_{AMPDIoU}=1-{IoU}^{\alpha }-{\left(\frac{d_{1}^{2}}{h^{2}+w^{2}}\right)}^{\alpha }-{\left(\frac{d_{2}^{2}}{h^{2}+w^{2}}\right)}^{\alpha }.$$

## 4. Datasets and Experimental Setup

### 4.1. Datasets

To validate our model, we used two datasets concurrently, allowing for a rigorous evaluation of the MRA-YOLOv8 network’s capabilities.

The first dataset is the photovoltaic electroluminescence anomaly detection (PVEL-AD) dataset [44]. This dataset is specifically designed for the task of PV cell defect detection, jointly released by Hebei University of Technology and Beihang University. It contains one class of non-anomalous images and 12 different categories of anomalous defect images.

The second dataset is the SPDI dataset, which is also designed for the task of PV cell defect detection, sourced from PaddlePaddle. It includes four categories of anomalous defect images: bird drop, clean, cracked, and dust.

For our experimental evaluation, we selected a subset of categories from each dataset based on annotation completeness and sample quantity. From the PVEL-AD dataset, we chose seven defect categories, including black core, line crack, finger interruption, star crack, thick line, horizontal dislocation, and short circuit, which have sufficient annotations and sample sizes to ensure reliable model training and evaluation. From the SPDI dataset, we selected three categories, including clean, cracked, and dust, that meet our criteria for annotation quality and sample quantity. The bird drop category was excluded due to an insufficient annotated sample for reliable model evaluation.

For both datasets, the data are divided into training, validation, and testing sets in a ratio of 7:2:1, ensuring a balanced and comprehensive evaluation of the model’s performance.

### 4.2. Experimental Setup

This study was conducted using the Ubuntu 22.04 operating system, with a 16 vCPU Intel(R) Xeon(R) Gold 6430 processor (Intel Corporation, Santa Clara, CA, USA) and an NVIDIA Geforce RTX 4090 (24 GB) graphics processing unit (NVIDIA Corporation, Santa Clara, CA, USA). The GPU-accelerated environment utilized CUDA 12.1, and the network architecture was implemented using Python 3.10 and PyTorch 2.1. To ensure that the proposed model achieved satisfactory results during training, we set the parameters as described below, based on relevant work and considering the characteristics of the various datasets. In this study, we used SGD as the optimizer for the model. The parameters were set as follows: the number of epochs was 300, the batch size was 8, the image size was 640 × 640, the initial learning rate was 0.01, the momentum was 0.937, the weight decay was 0.0005, and the warmup epochs were 3.

### 4.3. Evaluation Indicators

To better evaluate the model’s performance, we used the most commonly adopted metric in object detection tasks: precision (*P*), recall (*R*), F1-score (*F*1), mean average precision (*mAP*) [45], and Giga Floating Point Operations (*GFLOPs*). The calculation formula is as follows:

(25) $$P=\frac{TP}{TP+FP},$$

(26) $$R=\frac{TP}{TP+FN},$$

(27) $$F1=\frac{2\times P\times R}{P+R},$$

(28) $$AP=\int_{0}^{1}P(R)dR,$$

(29) $$mAP=\frac{1}{C}\sum_{i=1}^{C}{AP}_{i},$$

where *TP* represents the number of correctly predicted PV cell defects, *FP* denotes the number of incorrectly predicted defects, *FN* stands for the number of missed defects, and *C* is the number of classes. *GFLOPs* measure the theoretical computational cost of the model, calculated based on input resolution and network architecture.

## 5. Experimental Results and Analysis

### 5.1. Ablation Experiments

To precisely evaluate the effectiveness of the MBCANet, ResBlock, and AMPDIoU modules in the proposed MRA-YOLOv8 network, we conducted ablation experiments on the PVEL-AD and SPDI datasets. Below is a detailed analysis and discussion of the experimental results.

Table 1 shows the changes in model accuracy on the PVEL-AD dataset after gradually introducing the MBCANet, ResBlock, and AMPDIoU modules. As seen in the table, the inclusion of each module positively impacted the model’s performance, and the integration of multiple modules further enhanced the detection accuracy.

**Figure 4:**
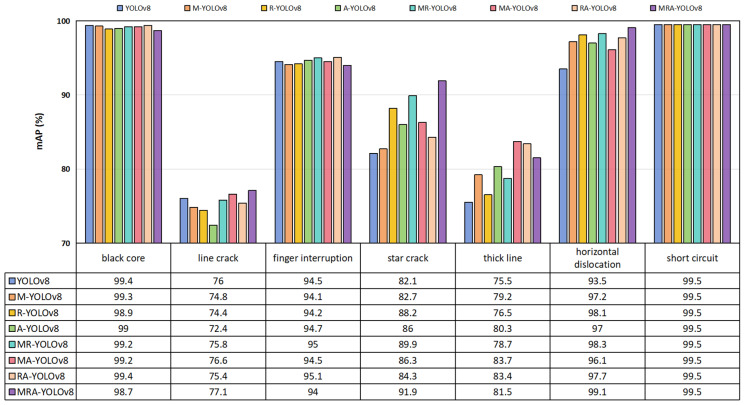
Ablation experiment results for defect categories such as black cores, line cracks, finger interruptions, and star cracks in the PVEL-AD dataset.

The introduction of the MBCANet increased the *mAP*_50_ by 0.9%, demonstrating the effectiveness of this module in extracting PV cell defect features. The MBCANet refines the network’s emphasis on deep features across horizontal and vertical spatial axes, allowing the model to detect PV cell defects more precisely. The ResBlock module, by introducing residual connections, increased the *mAP*_50_ by 1.4%. This highlights its superiority in enhancing the model’s ability to extract deep features, demonstrating its advantage in improving deep feature extraction capabilities. It effectively alleviated the issues of gradient vanishing and exploding during network training, enabling the model to learn richer deep feature information. The adoption of the AMPDIoU loss function increased the *mAP*_50_ by 1.2%, proving its effectiveness in optimizing object detection tasks. It reduced the discrepancy between the top-left and bottom-right coordinates of the predicted and actual bounding boxes. Additionally, combining the Alpha-IoU method improved the model’s precision in locating PV cell defects. By integrating different modules, we observed a further improvement in the model’s performance. Specifically, when the MBCANet, ResBlock, and AMPDIoU modules were introduced simultaneously, the *mAP*_50_ increased by 3.1%, reaching 91.7%. This result fully demonstrates the excellent performance of these three modules in the task of PV cell defect detection. Additionally, the inclusion of these modules also positively impacted other metrics such as precision, recall, F1-score, and *GFLOPs*, further validating their effectiveness in enhancing overall model performance. Figure 4 presents the ablation experiment results for each type of defect in the PVEL-AD dataset. As shown in the figure, compared to the YOLOv8 network, the MRA-YOLOv8 network exhibited the most significant improvements in detecting star crack, thick line, and horizontal dislocation defects in the PVEL-AD dataset. The *mAP*_50_ for these defect types increased by 9.8%, 6%, and 5.6%, respectively. The introduction of these modules not only enhanced the model’s feature extraction abilities but also optimized the loss function in the object detection task. The experimental results demonstrate the effectiveness and superiority of the MRA-YOLOv8 network, offering an efficient and precise solution for detecting PV cell defects.

The ablation experiments based on the SPDI dataset indicated that the MBCANet, ResBlock, and AMPDIoU modules significantly contributed to the performance enhancement of the YOLOv8 network. In this study, we kept the network’s base structure intact while testing the effects of these modules, both individually and in combination, on the model’s performance.

We employed ablation experiments to quantify the impact of each module on the performance of the YOLOv8 network using the SPDI dataset. Under controlled experimental conditions, we systematically evaluated the impact of these modules on the model’s *mAP*_50_ and *mAP*_50–95_ by introducing and integrating them individually. The experimental results, as demonstrated in Table 2, indicated that, whether added individually or in combination, the MBCANet, ResBlock, and AMPDIoU modules could effectively enhance the performance of the YOLOv8 network. Specifically, when the MBCANet or ResBlock module was added individually, the *mAP*_50_ improved, with the ResBlock module showing a more significant enhancement. While the introduction of the AMPDIoU module also increased the *mAP*_50_, there was a slight decline in the *mAP*_50–95_. When these modules were used in combination, the model’s performance improved further. Specifically, when the MBCANet, ResBlock, and AMPDIoU modules were introduced simultaneously, the model achieved optimal performance. The *mAP*_50_ increased by 2.1%, reaching 69.3%, and the *mAP*_50–95_ improved by 0.6%, reaching 52.7%. This result fully demonstrates the effectiveness of these modules in enhancing the capabilities of the YOLOv8 network. These modules improved the model’s feature extraction capabilities and optimized the loss function, thereby enhancing the model’s performance with the SPDI dataset. Specifically, the improvements in feature extraction provided by the MBCANet and ResBlock modules are essential in enhancing the model’s performance, while the AMPDIoU module further improves the model’s performance by optimizing the loss function. Additionally, on the SPDI dataset, we also observed that the inclusion of these modules positively impacted other metrics such as precision, recall, F1-score, and *GFLOPs*. We also noted that the *GFLOPs* value is dependent on the model structure rather than the dataset.

**Figure 5:**
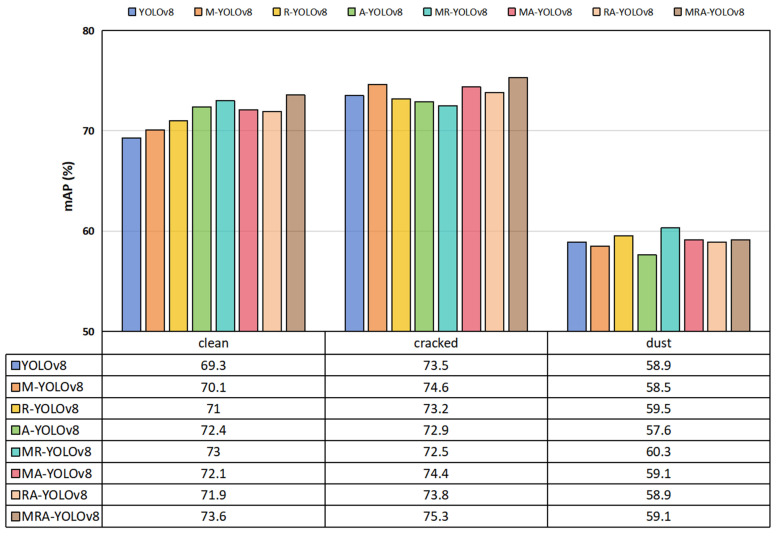
Ablation experiment results for the clean, cracked, and dust defect categories in the SPDI dataset, using *mAP*_50_ as the evaluation metric.

Figure 5 presents the ablation experiment results for the clean, cracked, and dust defects in the SPDI dataset. Firstly, by integrating the MBCANet, ResBlock, and AMPDIoU modules, the MRA-YOLOv8 model achieved higher detection accuracy across various defects, with accuracies of 73.6%, 75.3%, and 59.1% for clean, cracked, and dust defects, respectively. Specifically, for the clean defects, the MRA-YOLOv8, MR-YOLOv8, and R-YOLOv8 models performed well, with accuracies exceeding 70%, while the accuracies of the other models ranged between 69.3% and 72.4%. For cracked defects, the MRA-YOLOv8 and MA-YOLOv8 models performed the best, with accuracies exceeding 74%, while the accuracies of the other models ranged between 71% and 74.6%. Different modules exhibit varying effects on different types of defects. For example, the MBCANet and ResBlock modules performed well on clean and cracked defects, while the AMPDIoU module was relatively less effective on dust defects. Therefore, in practical applications, we can select the appropriate combination of modules based on specific requirements to optimize model performance.

### 5.2. Comparison Experiments

To further validate the performance of the proposed improvements in PV cell defect detection, we compared the MRA-YOLOv8 network with other models, including YOLOv3 [46], YOLOv5 [47], YOLOv6 [48], YOLOv8 [49], YOLOv9 [50], RetinaNet [51], ATSS [52], YOLOX Tiny [53], detection transformer (DETR) [54], and AutoAssign [55], using the PVEL-AD and SPDI datasets. A comprehensive analysis of the detection results was then conducted.

In the comparative experiments using the PVEL-AD dataset, the proposed MRA-YOLOv8 model demonstrated outstanding performance in PV cell defect detection tasks, as summarized in Table 3. Specifically, the model achieved a significant advantage in the *mAP* metric, with a *mAP*_50_ reaching 91.7%, which is notably higher than that of the other models. Compared to the well-performing YOLOv6, YOLOv8, and YOLOv9 models, the MRA-YOLOv8 achieved a *mAP*_50_ improvement of 3.4%, 3.1%, and 1.6%. The model not only excelled in *mAP*_50_ but also outperformed all other models in the *mAP*_50–95_ metric, achieving a score of 62%. This indicates that MRA-YOLOv8 can maintain excellent detection performance across different IoU thresholds, further validating its effectiveness and robustness in object detection tasks. Additionally, the proposed model showed superior performance in other key metrics, such as precision, recall, and F1-score, which are positively correlated with *mAP*_50_. The MRA-YOLOv8 achieved a precision of 89%, recall of 86.5%, and F1-score of 87.7%, further highlighting its balanced and robust detection capabilities. Moreover, the model’s *GFLOPs* value of 10 is relatively low compared to other high-performing models like YOLOv9, demonstrating an excellent balance between computational efficiency and detection accuracy.

**Figure 6:**
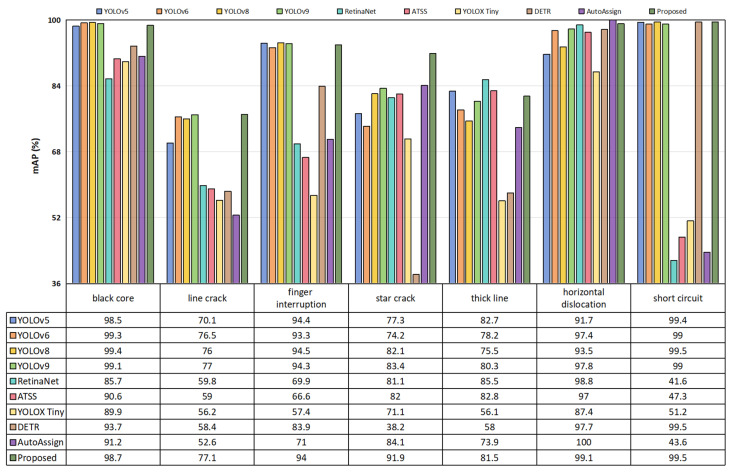
Comparison experiment results for defect categories such as black cores, line cracks, finger interruptions, and star cracks in the PVEL-AD dataset.

Additionally, we analyzed each defect type in the PVEL-AD dataset, with the results presented in Figure 6. As shown in the figure, the proposed MRA-YOLOv8 network effectively detected all defect types in the PVEL-AD dataset. Particularly for the detection of star crack defects, the *mAP*_50_ reached 91.9%, significantly higher than other models. Compared to YOLOv8, the *mAP*_50_ increased by 9.8%.

Table 4 displays the test results for the SPDI dataset. The YOLO series consistently demonstrated high accuracy in the field of object detection. According to the experimental results, the YOLO series performed excellently in both the *mAP*_50_ and *mAP*_50–95_ metrics. Among them, YOLOv9 achieved a *mAP*_50_ of 68.3%, while YOLOv8 reached a *mAP*_50–95_ of 52.1%. These results indicate that the YOLO series possesses high accuracy in handling objects of varying scales and shapes. However, the MRA-YOLOv8 achieves a *mAP*_50_ of 69.3%, which is 1% higher than the best-performing model in the YOLO series, YOLOv9. In addition to the YOLO series, we also compared the performance of the ATSS, YOLOX Tiny, DETR, and AutoAssign models. These models generally scored lower than the YOLO series in both *mAP*_50_ and *mAP*_50–95_ metrics and were unable to match the performance of MRA-YOLOv8. Notably, AutoAssign shows better performance for horizontal dislocation compared to other models, which is an important consideration in certain detection scenarios. ATSS achieved a *mAP*_50_ of only 42.2%, which is 27.1% lower than MRA-YOLOv8. Additionally, ATSS also scored significantly lower than MRA-YOLOv8 in the *mAP*_50–95_ metric. In the comparative experiments on the SPDI dataset, we also observed that the inclusion of these modules positively impacted other metrics such as precision, recall, F1-score, and *GFLOPs*.

**Figure 7:**
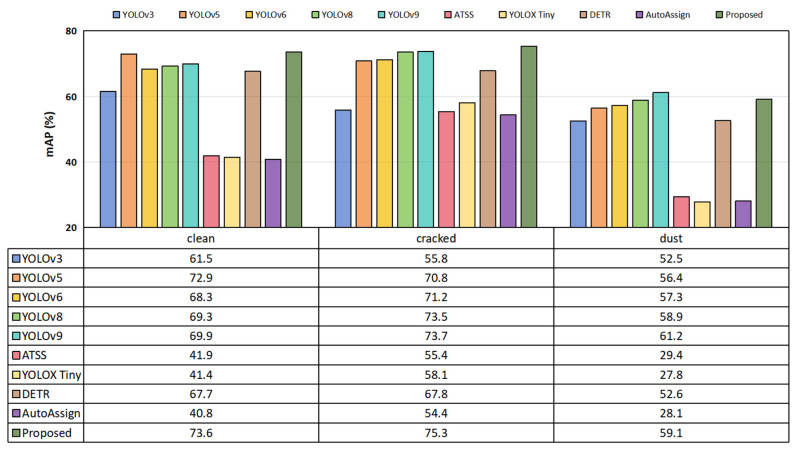
Comparison experiment results for the clean, cracked, and dust defect categories in the SPDI dataset, using *mAP*_50_ as the evaluation metric.

We performed a detailed analysis of each defect type in the SPDI dataset. The results are depicted in Figure 7. As shown in the figure, the proposed MRA-YOLOv8 exhibited strong performance in detecting each type of defect in the SPDI dataset. For the clean defect, MRA-YOLOv8 achieved a *mAP*_50_ of 73.6%, significantly surpassing YOLOv3’s 61.5% and YOLOv6’s 68.3%. This indicates that MRA-YOLOv8 can more accurately detect and localize targets in ideal, unobstructed image environments. Furthermore, when dealing with cracked defects, the structural integrity and performance of the target are compromised, posing a greater challenge to detection algorithms. However, MRA-YOLOv8 still achieved a *mAP*_50_ of 75.3%, significantly higher than YOLOv3’s 55.8% and YOLOX Tiny’s 58.1%, demonstrating its outstanding robustness and adaptability. For dust defects, the visibility and contrast of the image were typically low, further increasing the detection difficulty. Nonetheless, MRA-YOLOv8 achieved a *mAP*_50_ of 59.1%, outperforming most detection algorithms.

### 5.3. Comparison of the Performance Between MBCANet and Other Attention Networks

To assess the MBCANet’s effectiveness, we compared it with other attention networks, including SENet [56], CBAM [57], EMANet [58], and CANet.

On the basis of integrating the ResBlock and AMPDIoU modules, we placed the four mainstream networks, SENet, CBAM, EMANet, and CANet, along with our proposed MBCANet, in the backbone for feature extraction. We then compared their performance with the PVEL-AD and SPDI datasets.

Table 5 lists the *mAP*_50_ and *mAP*_50–95_ of the MBCANet and other attention networks using the PVEL-AD dataset. As shown in the table, the MBCANet achieved the best performance in both metrics, with a *mAP*_50_ of 91.7% and a *mAP*_50–95_ of 62%. Compared to the other models, the MBCANet outperformed SENet, CBAM, EMANet, and CANet in *mAP*_50_ by 1.1%, 1.4%, 1.5%, and 1.8%, respectively. In *mAP*_50–95_, the MBCANet was higher by 1.2%, 0.4%, 0.6%, and 1%, respectively. These results fully demonstrate the effectiveness and superiority of the MBCANet in PV cell defect detection tasks.

**Figure 8:**
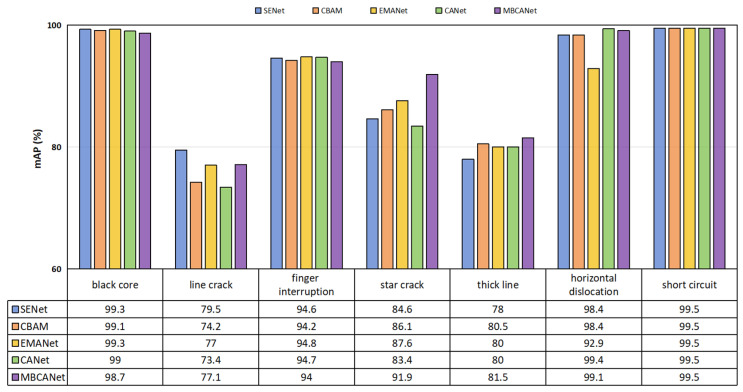
Comparison results of MBCANet and other attention networks for defect categories such as black cores and line cracks in the PVEL-AD dataset.

Additionally, we analyzed each type of defect in the PVEL-AD dataset, with the results presented in Figure 8. As shown in the figure, the proposed MBCANet performed well in detecting each type of defect in the PVEL-AD dataset. For detecting star crack defects, the *mAP*_50_ reached 91.9%, significantly higher than the other models. This is due to its multi-branch design, which effectively addresses the gradient vanishing problem. In contrast, while other attention networks may have performed better on certain specific defect categories, in conclusion, they were not as consistent as the MBCANet.

The test results for the SPDI dataset are shown in Table 6. It can be seen from the table that the MBCANet achieved a significant performance advantage with the SPDI dataset. Specifically, the MBCANet scored 69.3% in the *mAP*_50_ metric, showing an improvement over other attention networks. In the *mAP*_50–95_ metric, although EMANet scored slightly higher than the MBCANet, the difference between the two was minimal. The MBCANet’s performance remained superior compared to the other models.

As shown in Table 7, the MBCANet achieved good performance on each type of defect in the SPDI dataset. Specifically, for the clean category, the MBCANet achieved a *mAP*_50_ of 73.6%, slightly higher than the other attention networks. For the cracked category, the MBCANet’s *mAP*_50_ reached 75.3%, significantly outperforming the other models. For the dust category, although the MBCANet did not achieve the highest *mAP*_50_, the gap between this algorithm and the best-performing SENet is minimal. In summary, the MBCANet demonstrated strong performance across various defect types in the SPDI dataset, particularly excelling in the cracked category. Compared to other advanced attention networks, the MBCANet has advantages in feature extraction and attention mechanism design, enabling it to more effectively capture and utilize relevant features, thus achieving the accurate detection of various defects.

### 5.4. Comparison of the Performance of AMPDIoU and Other Loss Functions

To validate the effectiveness of AMPDIoU, we compared it with other loss functions, including GIoU, DIoU [59], SIoU [60], EIoU [61], and Focal- EIoU.

Building on the integration of the MBCANet and ResBlock modules, we compared the performance of the five loss functions with our proposed AMPDIoU using the datasets.

As shown in Table 8, AMPDIoU achieved the best results in both *mAP*_50_ and *mAP*_50–95_ metrics. Specifically, AMPDIoU achieved a *mAP*_50_ of 91.7%, surpassing all other loss functions. In the *mAP*_50–95_ metric, AMPDIoU also achieved an accuracy of 62%, providing a distinct advantage over other loss functions. This is primarily because AMPDIoU enhances the precision and robustness of small object detection by integrating the methods of MPDIoU and Alpha-IoU.

**Figure 9:**
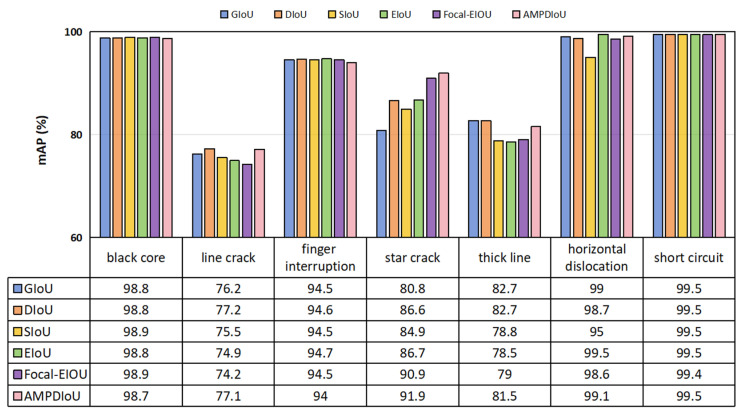
Comparison results of AMPDIoU and other loss functions for defect categories such as black cores and line cracks in the PVEL-AD dataset.

Additionally, we analyzed each type of defect in the PVEL-AD dataset, with the results shown in Figure 9. The figure shows that the proposed AMPDIoU performed well in detecting each type of defect in the PVEL-AD dataset. For the detection of star crack defects, the *mAP*_50_ reached 91.9%, markedly superior to the other models. Compared to GIoU, DIoU, SIoU, EIoU, and Focal-EIoU, the *mAP*_50_ improved by 11.1%, 5.3%, 7%, 5.2%, and 1%, respectively.

The test results for the SPDI dataset are presented in Table 9. As shown in the table, we conducted detailed performance comparison experiments between the AMPDIoU loss function and several other popular loss functions using the SPDI dataset. In terms of the key metric *mAP*_50_, AMPDIoU significantly outperformed the other loss functions. Specifically, AMPDIoU achieved a *mAP*_50_ of 69.3%, which represents an improvement of 0.9% over GIoU, 1.7% over DIoU, 2% over SIoU, 0.9% over EIoU, and 0.4% over Focal-EIoU. These results indicate that AMPDIoU can accurately predict the position and size of targets. The *mAP*_50–95_ for AMPDIoU was 52.7%, similar to DIoU, EIoU, and Focal-EIoU, but slightly higher than GIoU and SIoU, demonstrating the superior performance of AMPDIoU. Further analysis revealed that the exceptional performance of AMPDIoU with the SPDI dataset was mainly due to its unique design. AMPDIoU considers multiple factors in the loss function, including the center point distance and boundary regression, allowing the model to better adapt to different shapes and sizes of target detection tasks. In contrast, other loss functions may have limitations in certain aspects, leading to poorer performance in handling complex targets.

Simultaneously, we analyzed each type of defect in the SPDI dataset, with the results presented in Table 10. As shown in the table, the proposed AMPDIoU demonstrated considerable competitiveness in the *mAP*_50_ metric for different types of defects. Notably, in the detection of cracked defects, AMPDIoU demonstrated a *mAP*_50_ value of 75.3%, significantly higher than the other loss functions. This indicates that AMPDIoU has higher accuracy in handling small-sized defects. Furthermore, compared to GIoU, DIoU, SIoU, EIoU, and Focal-EIoU, AMPDIoU maintained an advantage or comparable levels in the detection of clean and dust defects, reflecting its generalization capability and stability.

### 5.5. Experimental Analysis of MBCANet Branch Contributions

To investigate the independent contributions and synergistic effects of each branch in the MBCANet for PV cell defect detection, this study conducted isolated tests on the performance of its three branches using the PVEL-AD and SPDI datasets. By comparing the precision, recall, F1-scores, and computational efficiency between single-branch and full-branch configurations, the experiments revealed the strengths and weaknesses of different feature extraction strategies and the potential for combined optimization.

In the PVEL-AD dataset, Branch 2 achieved the best performance with an F1-score of 85.3% and *mAP*_50–95_ of 61.7%, as detailed in Table 11, demonstrating that its multi-directional feature fusion significantly enhanced defect localization robustness. While Branch 1 led in precision, its recall was limited. Branch 3 stood out in terms of balanced performance. After integrating all branches, the model’s overall performance further improved, and the computational cost did not increase significantly, validating the efficiency of multi-branch collaboration.

In the SPDI dataset, as shown in Table 12, while the integration of all branches resulted in a slight reduction in precision, it increased the *mAP*_50–95_ to 53% and maintained stable GFLOPs. This demonstrates that multi-directional feature modeling effectively balances task requirements with computational efficiency. The experimental results confirm that the MBCANet achieves an optimal balance between accuracy and efficiency through lightweight branch design and residual structures.

### 5.6. Visual Presentation

**Figure 10:**
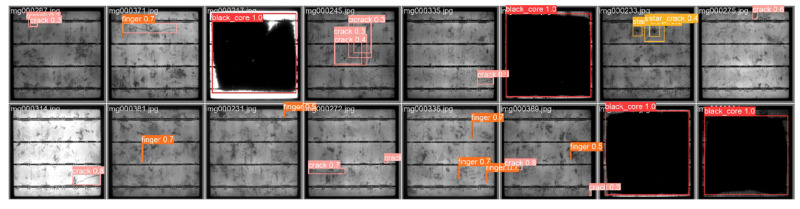
Visualization results for the PVEL-AD dataset. The 16 images shown were randomly selected and include various defect categories.

Figure 10 presents the visualization results of our proposed MRA-YOLOv8 network using the PVEL-AD dataset, demonstrating the network’s effectiveness and accuracy in identifying and classifying PV cell defects. As shown in the figure, the MRA-YOLOv8 network successfully identified and classified various types of defects, including cracks, finger defects, black cores, and star cracks. Additionally, the MRA-YOLOv8 network detected multiple defects within a single PV cell image, showcasing its multi-task processing capabilities. Notably, the model remained capable of detecting defects under varying lighting and background conditions, even in images with significant background noise. This indicates the model’s robust performance.

From this analysis, it is evident that the MRA-YOLOv8 network excels in PV cell defect detection tasks, offering high-precision, multi-type detection, multi-task processing capabilities, and strong robustness.

**Figure 11:**
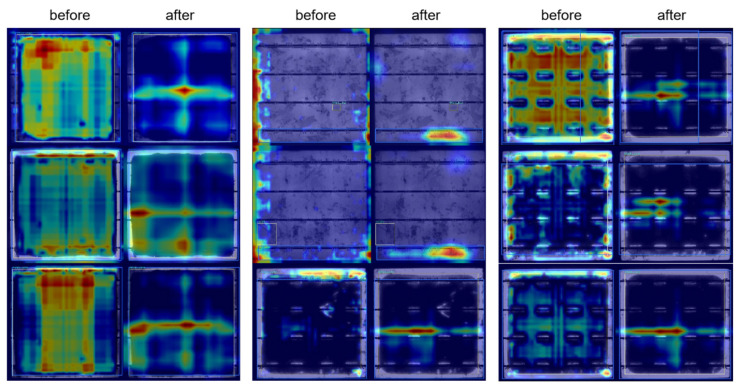
Heatmaps of MBCANet on the PVEL-AD dataset.

Figure 11 presents attention heatmaps of the MBCANet on the PVEL-AD dataset, visually illustrating its ability to suppress background noise by focusing on critical regions. In the heatmaps without the MBCANet, the attention distribution is scattered, with significant activation in non-target background areas, which may result in reduced precision for detecting small defects. In contrast, the heatmaps with the MBCANet demonstrate a more concentrated attention pattern on the target regions, effectively reducing background interference. These results indicate that the MBCANet consistently enhances the model’s focus on small-scale defects across diverse and cluttered industrial scenarios, thereby improving detection accuracy and robustness.

### 5.7. Discussion

The experimental results validated the effectiveness of the MRA-YOLOv8 framework in achieving high-precision PV cell defect detection across complex industrial scenarios. On the PVEL-AD dataset, the model attained a *mAP*_50_ of 91.7%, outperforming advanced methods like YOLOv9 while maintaining computational efficiency. For the SPDI dataset, it achieved a competitive *mAP*_50_ of 69.3%, demonstrating robust generalization despite challenges such as background clutter and low-contrast defects like dust contamination.

Central to this performance were the synergistic contributions of the MBCANet, ResBlock, and AMPDIoU. The attention heatmaps revealed that the MBCANet suppressed background noise by concentrating on critical defect regions, particularly enhancing the detection of small-scale defects. ResBlock alleviated gradient vanishing issues and strengthened deep feature extraction, while AMPDIoU optimized bounding box regression through a fusion of MPDIoU and Alpha-IoU, surpassing GIoU and DIoU in both localization accuracy and robustness. This integration ensured precise defect localization even under noisy conditions, as evidenced by the 3.1% *mAP*_50_ gain when all modules were combined.

Compared to existing methods, MRA-YOLOv8 struck a unique balance between accuracy and efficiency. While one-stage detectors like YOLOv4 prioritized speed, their precision lagged significantly. Conversely, two-stage approaches like Faster R-CNN achieved higher accuracy but at the cost of computational overhead. The proposed model bridged this gap, offering real-time applicability without sacrificing detection quality—a critical advantage for industrial deployment.

However, limitations persisted. A slight precision decline on the SPDI dataset suggested potential overfitting when integrating all modules under limited annotation diversity. Additionally, the model’s reliance on high-quality public dataset annotations could hinder adaptability to highly customized industrial environments with unique defect patterns. Future work should explore lightweight architectures for edge-device compatibility and semi-supervised learning to reduce annotation dependency.

In conclusion, MRA-YOLOv8 advanced PV defect detection by harmonizing innovative module design with practical efficiency. Its success in diverse industrial settings underscored its potential as a versatile tool for automated quality inspection, with broader applicability to manufacturing defect detection tasks. Addressing current limitations through adaptive training and multi-sensor fusion would further solidify its role in smart industrial systems.

## 6. Conclusions and Future Work

Due to the complex backgrounds and the low proportion of defect feature information in PV cell images, we propose a new PV cell defect detection method, MRA-YOLOv8. First, a multi-branch coordinate attention network (MBCANet) was introduced into the backbone. The incorporation of a CANet aims to mitigate the noise impact of background information on the detection task, while the use of multiple branches enhances the model’s feature extraction capability. Second, we integrated a multi-path feature extraction module (ResBlock) into the neck. This module provides finer-grained multi-scale features, improving feature extraction from complex backgrounds and enhancing the model’s robustness. Finally, we applied AMPDIoU to the head, combining the methods of MPDIoU and Alpha-IoU. The experimental results showed that, with the PVEL-AD dataset, this method achieved a *mAP*_50_ of 91.7%, an improvement of 3.1% over YOLOv8 and 16.1% over DETR. With the SPDI dataset, it achieved a *mAP*_50_ of 69.3%, an improvement of 2.1% over YOLOv8 and 6.6% over DETR.

In summary, although this method achieves the precise identification of PV cell defects, it still has certain limitations. The lower number of certain categories in the dataset results in lower learning accuracy for those targets, which, in turn, affects the overall detection accuracy. For example, in the PVEL-AD dataset, the star crack defect category accounts for only 1.76% of the total samples, with an imbalance ratio of 21.91. Similarly, in the SPDI dataset, the dust defect category represents only 17.21% of the samples, with an imbalance ratio of 2.96. To eliminate biases in the detection results, the dataset needs further improvement. Additionally, the datasets utilized in this study focus on silicon-based PV cells. To enhance the model’s generalization capabilities, a more in-depth exploration of different types of PV cells is necessary. Therefore, in future work, we will conduct the following studies: (1) exploring the generation of more realistic datasets; (2) lightweighting the model for deployment on detection devices; (3) using generative adversarial networks (GANs) to generate synthetic defect data; and (4) fine-tuning for different types of PV cell datasets to further enhance the model’s generalization performance.

## Figures and Tables

**Table 1:** Overall ablation experiment results for the PVEL-AD dataset.

Original	MBCANet	ResBlock	AMPDIoU	*P*	*R*	*F*1	*mAP*_50_	*mAP*_50–95_	*GFLOPs*
√				85.2	83.5	84.3	88.6	60.5	8.1
√	√			86.1	84.7	85.4	89.5	61	8.2
√		√		85.7	83.8	84.7	90	61.2	10
√			√	86.3	83	84.6	89.8	61.1	8.1
√	√	√		87	86.9	86.9	90.9	61.6	10
√	√		√	90.9	80.2	85.2	90.8	61.5	8.2
√		√	√	88.6	82.5	85.4	90.7	61.7	10
√	√	√	√	89	86.5	87.7	91.7	62	10

**Table 2:** Overall ablation experiment results for the SPDI dataset.

Original	MBCANet	ResBlock	AMPDIoU	*P*	*R*	*F*1	*mAP*_50_	*mAP*_50–95_	*GFLOPs*
√				76.4	61	67.8	67.2	52.1	8.1
√	√			78.8	59.6	67.9	67.7	53	8.2
√		√		75.2	64.1	69.2	67.9	52.9	10
√			√	75.6	60.3	67.1	67.6	51.5	8.1
√	√	√		75	64.1	69.1	68.6	52.9	10
√	√		√	73.9	63.3	68.2	68.5	53.3	8.2
√		√	√	78.4	61	68.6	68.2	52.1	10
√	√	√	√	78.1	63	69.7	69.3	52.7	10

**Table 3:** Overall comparison experiment results for the PVEL-AD dataset.

Model	*P*	*R*	*F*1	*mAP*_50_	*mAP*_50–95_	*GFLOPs*
YOLOv5	84.1	82.6	83.3	87.7	59.4	7.1
YOLOv6	84.6	83	83.8	88.3	60.1	11.4
YOLOv8	85.2	83.5	84.3	88.6	60.5	8.1
YOLOv9	89.5	82.1	85.6	90.1	61.5	102.4
RetinaNet	-	-	-	74.6	44.1	-
ATSS	-	-	-	75	40.4	-
YOLOX Tiny	-	-	-	67	35.9	6.5
DETR	71.9	69.9	70.9	75.6	52.7	30.3
AutoAssign	-	-	-	73.8	42	-
Proposed	89	86.5	87.7	91.7	62	10

**Table 4:** Overall comparison experiment results for the SPDI dataset.

Model	*P*	*R*	*F*1	*mAP*_50_	*mAP*_50–95_	*GFLOPs*
YOLOv3	57.5	58.9	58.2	56.6	33.7	5.9
YOLOv5	69.3	64.3	66.7	66.7	50.7	7.1
YOLOv6	73.8	59.1	65.6	65.6	49.9	11.4
YOLOv8	76.4	61	67.8	67.2	52.1	8.1
YOLOv9	83.3	61.8	71	68.3	53.1	102.4
ATSS	-	-	-	42.2	32.4	-
YOLOX Tiny	-	-	-	42.4	32.9	6.5
DETR	72.2	62	66.7	62.7	49.3	30.3
AutoAssign	-	-	-	41.1	31.8	-
Proposed	78.1	63	69.7	69.3	52.7	10

**Table 5:** Overall comparison results of MBCANet and other attention networks using the PVEL-AD dataset.

	*mAP*_50_	*mAP*_50_ Gap	*mAP*_50–95_	*mAP*_50–95_ Gap	*GFLOPs*
SENet	90.6	−1.1	60.8	−1.2	10
CBAM	90.3	−1.4	61.6	−0.4	10.2
EMANet	90.2	−1.5	61.4	−0.6	10
CANet	89.9	−1.8	61	−1	10
MBCANet	91.7	-	62	-	10

**Table 6:** Overall comparison results of MBCANet and other attention networks using the SPDI dataset.

	*mAP*_50_	*mAP*_50_ Gap	*mAP*_50–95_	*mAP*_50–95_ Gap	*GFLOPs*
SENet	68.5	−0.8	51.7	−1	10
CBAM	67.3	−2	50.3	−2.4	10.2
EMANet	68.4	−0.9	53.1	+0.4	10
CANet	67.7	−1.6	51.9	−0.8	10
MBCANet	69.3	-	52.7	-	10

**Table 7:** Comparison results of MBCANet and other attention networks for each defect category in the SPDI dataset.

	Clean	Cracked	Dust
SENet	71.3	71.1	63.2
CBAM	66.5	73.3	62
EMANet	71.5	73.3	60.4
CANet	73.2	72	57.9
MBCANet	73.6	75.3	59.1

**Table 8:** Overall comparison results of AMPDIoU and other loss functions using the PVEL-AD dataset.

	*mAP*_50_	*mAP*_50_ Gap	*mAP*_50–95_	*mAP*_50–95_ Gap	*GFLOPs*
GIoU	90.2	−1.5	61.8	−0.2	10
DIoU	91.2	−0.5	61.8	−0.2	10
SIoU	89.6	−2.1	61.5	−0.5	10
EIoU	90.4	−1.3	61.4	−0.6	10
Focal-EIoU	90.8	−0.9	62	0	10
AMPDIoU	91.7	-	62	-	10

**Table 9:** Overall comparison results of AMPDIoU and other loss functions using the SPDI dataset.

	*mAP*_50_	*mAP*_50_ Gap	*mAP*_50–95_	*mAP*_50–95_ Gap	*GFLOPs*
GIoU	68.4	−0.9	51.7	−1	10
DIoU	67.6	−1.7	52.8	+0.1	10
SIoU	67.3	−2	50.3	−2.4	10
EIoU	68.4	−0.9	52.6	−0.1	10
Focal-EIoU	68.9	−0.4	52.9	+0.2	10
AMPDIoU	69.3	-	52.7	-	10

**Table 10:** Comparison results of AMPDIoU and other loss functions for each defect category in the SPDI dataset.

	Clean	Cracked	Dust
GIoU	74.5	71.4	59.4
DIoU	71.2	73.9	57.6
SIoU	74.1	71.7	56.2
EIoU	73.6	74	57.5
Focal-EIoU	71.8	76.3	58.5
AMPDIoU	73.6	75.3	59.1

**Table 11:** Test results of isolating the three branches of MBCANet on the PVEL-AD dataset.

	*P*	*R*	*F*1	*mAP*_50_	*mAP*_50–95_	*GFLOPs*
Branch 1	87.6	80.6	84	89.1	60.3	8.2
Branch 2	87.1	83.6	85.3	89.2	61.7	8.1
Branch 3	85.2	83.5	84.3	88.6	60.5	8.1
All	86.1	84.7	85.4	89.5	61	8.2

**Table 12:** Test results of isolating the three branches of MBCANet on the SPDI dataset.

	*P*	*R*	*F*1	*mAP*_50_	*mAP*_50–95_	*GFLOPs*
Branch 1	80.2	62.5	70.3	67.6	51.9	8.2
Branch 2	79.2	61	68.9	67.5	52.3	8.1
Branch 3	76.4	61	67.8	67.2	52.1	8.1
All	78.8	59.6	67.9	67.7	53	8.2

## Data Availability

The original contributions presented in this study are included in the article; further inquiries can be directed to the corresponding authors.
